# Calcium - Magnesium imbalance implicated in benign prostatic hyperplasia and restoration by a phytotherapeutic drug – *Croton membranaceus* Müll.Arg

**DOI:** 10.1186/s12906-017-1663-x

**Published:** 2017-03-11

**Authors:** George Awuku Asare, Robert A. Ngala, Daniel Afriyie, Samuel Adjei, Adriana Nyarko, Yvonne Anang-Quartey, Bernice Asiedu, Derek Doku, Brodrick Y. Amoah, Kennedy Bentum, Iddi Musah, Kensese Mossanda

**Affiliations:** 1 University of Ghana School of Biomedical and Allied Health Sciences (SBAHS), Korle Bu, Ghana; 2 Department of Molecular Medicine, School of Medical Sciences, Kwame Nkrumah University of Science and Technology, Kumasi, Ghana; 3 Department of Pharmacy, Ghana Police Hospital, Cantonments, Accra, Ghana; 4 Noguchi Memorial Institute for Medical Research (NMIMR), University of Ghana, Legon, Ghana; 5 Department of Urology, Ghana Police Hospital, Cantonments, Accra, Ghana; 6 Directorate of Research, Walter Sisulu University, Mthatha, South Africa; 7 Chemical Pathology Unit, Department of Medical Laboratory Sciences, School of Biomedical and Allied Health Sciences, College of Health Sciences, University of Ghana, P.O. Box KB 143, Korle-bu, Accra, Ghana

**Keywords:** Calcium, Magnesium, Benign prostatic hyperplasia, *Croton membranaceus*

## Abstract

**Background:**

Calcium (Ca)- magnesium (Mg) imbalance is implicated in prostate cancer. Ca/Mg ratio increases or decreases with proliferation or apoptosis, respectively. The study examined whether this Ca/Mg imbalance exists in BPH patients and the effect of a phytotherapeutic drug on the Ca/Mg ratio.

**Methods:**

Thirty (30) BPH patients who used the ethanolic root extract of *Croton membranaceus* (60 mg/day) for 3 months were examined for serum Ca, Mg, phosphate, parathyroid hormone (PTH), vitamin D, prostate specific antigen (PSA) levels and renal function tests (RFT) before (BT) and after treatment (AT) alongside thirty (30) controls. Twenty (20) trace element including Mg and Ca were determined in the drug by neutron activation analysis (NAA).

**Results:**

RFT, PTH and vitamin D for BT, AT and controls (C) were normal. Mean PSA was 1.0 ± 0.64 (C), 27.9 ± 19.0 (BT) and 16.2 ± 11.8 ng/mL (AT) (*p* = 0.002). Mg, Ca/Mg ratio BT, AT and control were significantly different (*p* = 0.0001, respectively). After treatment, Mg and Ca/Mg ratio were not different from controls. The prevalence of Ca/Mg imbalance was 80% (BT), 13.3% (AT) and 3.3% (control group).

**Conclusion:**

Ca/Mg ratio imbalance is associated with BPH. This has previously not been demonstrated. The imbalance was significantly corrected after treatment with the phytotherapeutic drug.

## Background

Epidemiological studies of a cohort in Taiwan associated drinking water Ca and Mg levels, to prostate cancer (PCa) deaths. The authors concluded that Mg from the drinking water or other dietary sources was protective against prostate cancer [1]. Major trace elements such as cadmium (Cd), nickel (Ni), zinc (Zn), copper (Cu), iron (Fe), magnesium (Mg) and calcium (Ca) have previously been identified in benign and malignant prostate samples. The concentrations of Ca and Mg have been found to be significantly higher in the malignant prostate compared to the benign among other metals [2]. X-ray microanalysis on freeze-dried cryosections have been used to demonstrate that Ca in secretory vessels is several folds higher than Mg or Zn, buttressing the fact that Ca is the major prostate acinar cell cation [3]. Furthermore, within the cytoplasm a positive correlation between Ca and Mg as well as Ca and Zn in hyperplastic and normal glands has been demonstrated. Additionally, increasing intranuclei Ca with advancing age has been observed and is believed to have pathologic significance in prostate growth disorders [3]. At the cellular level studies strongly suggest the presence of Ca and Mg imbalance in PCa cases [4].

Elevated extracellular calcium is thought to prevent apoptosis via the Ca-sensing receptor [5]. Serum Mg levels of PCa patients when examined revealed a significantly high Ca/Mg ratio. This was accounted for by an equally significant lower Mg level. The odds indicated that an elevated Ca/Mg ratio was associated with the increased risk of high-grade PCa. On the other hand, elevated Mg was significantly associated with a lower risk. However, serum levels of Ca alone were not associated with PCa and Ca/Mg levels were also not associated with BPH [6]. The Ca/Mg ratio has been implicated in proliferation of prostate cancer cells compared to controls [4]. Ca^2+^ signaling is therefore important in regulating other physiological functions such as cell proliferation and differentiation in conjunction with Mg [6–10].

Mg upon mitotic stimulation can initiate cell proliferation by activating Mg^2+^ influx and increasing intracellular Mg^2+^ [11, 12]. Because both Ca and Mg are able to operate in a similar mechanism the efflux of both extracellular metals needs tight control. An increase in cytosolic Ca^2+^ for example regulates apoptosis [13, 14]. At the extracellular level, an increase in Ca^2+^ or a decrease in extracellular Mg^2+^ further increased Ca^2+^ influx. The efflux control of Ca and Mg is regulated by a melastatin-like transient receptor potential (TRPM). These are a diverse group of voltage-independent Ca^2+^-permeable cation channels present in mammalian cells. TRPM6/7 gene mutations have been demonstrated in hereditary hypomagnesaemia caused by Mg^2+^ reabsorption impairment [15]. Additionally, other studies have shown Mg^2+^ entry preference over Ca^2+.^ However, in the absence of Mg^2+^, the channels are able to conduct Ca^2+^ currents. Therefore, an increase in extracellular Ca^2+^ or a decrease in extracellular Mg^2+^ increases Ca^2+^ influx. Additionally, the subsequent increase in the Ca^2+^/Mg^2+^ ratio and TRPM7 expression has been demonstrated in age-matched prostate cancer patients. Therefore, an increase in the serum Ca^2+^/Mg^2+^ ratio will increase Ca^2+^ entry by the activation of TRPM7 channels, which eventually leads to increased cell proliferation and cancer formation [4].

Current literature has provided evidence of the Ca/Mg hemostasis and its involvement in cell proliferation as well as prostate cancer development. The tight regulation of this channel has also been attributed to the TRMP7 gene and the Ca/Mg ratio has also been demonstrated in clinical studies of PCa patients. However, what remains largely unknown is the presence or absence of this ratio imbalance in BPH patients. This study therefore sought to examine retrospectively, the Ca/Mg balance in a cohort of BPH patients before and after three (3) months of treatment.

## Methods

### Study design and site

The study was a retrospective observational study. The folders of 30 BPH patients under medical supervision who had opted for the use of a phytotherapeutic drug from the ethanolic root extract of *Croton membranaceus* capsules (20 mg t.i.d.) for a period of 3 months at the Ghana Police Hospital Urology Clinic were examined. Frozen archival blood samples were selected and examined based on patients who chose this form of therapy which had just been introduced between January 2013 and December 2014. The Ghana Police Hospital is one of the 11 centers in the country approved by the Ministry of Health to administer plant medicine. Thirty (30) other control subjects within the age group who were diagnosed negative for BPH within the study period were also used.

### Ethical issues

The project was approved by the Ethics and Protocol Review Committee of the School of Biomedical and Allied Health Sciences with Ethics number SAHS-ET/SAHS/PSM/ML/09/AA/26A/2012-2013. Furthermore, approval was sought from the Police Hospital administration. Informed consent was also sought from all participants whose information and samples were used. The study complied with the Helsinki Declaration of 1964, with revision in October 2008.

### Blood samples

A previous study was undertaken by this same group of researchers that demonstrated the efficacy of *Croton membranaceus* for BPH [16]. The rest of the samples were achieved for this study. The ethics approval of the previous study covered the present study. Archival blood samples from that previous study were selected and examined based on patients who chose this form of drug therapy (phytotherapy). Samples of patients who reported to the Urology Department between January 2013 and December 2014 and opted for the phototherapeutic treatment were used. All blood samples for the PSA test as requested by the urologist at that time were taken between 8 am and 12 pm. Blood samples (sera) were then stored in the -20 °C freezer. These samples were retrieved in January 2016 for the present study from the Biochemistry Laboratory freezer and analyzed for PSA, serum Ca, Mg, P, PTH, vitamin D, renal function (and albumin for calcium correction). The afore-mentioned assays were analyzed for single samples [control (C)] whilst test cases had analysis done for “before treatment” (BT) and “after treatment” (AT) samples.

### Phytotherapeutic drug trace element (TE) analysis

Twenty (20) trace elements of the phytotherapeutic drug [arsenic (As), chloride (Cl), cobalt (Co), chromium (Cr), copper (Cu), cadmium (Cd), mercury (Hg), lead (Pb), nickel (Ni), iron (Fe), manganese (Mn), iodine (I), vanadium (V), aluminum (Al), magnesium (Mg), selenium (Se), potassium (K), sodium (Na), calcium (Ca), and zinc (Zn)] were also examined by neutron activation analysis.

### Biochemical assays

#### PSA

Total PSA (TPSA) was performed using AccuBind total PSA ELISA kits purchased from MonoBind Inc. (California, USA) according to the manufacturer’s instructions. The ELISA plate was coated with highly specific monoclonal anti-PSA antibodies. In brief, 25 μl of samples together with standards were aliquoted into designated wells and incubated with 100 μl antibody-HRP enzyme conjugate at room temperature for 30 min. The plate was then washed with a wash buffer after which 100 μl of 3,3′,5,5′- tetramethylbenzidine (TMB)/hydrogen peroxide (substrate) was then added to react with the HRP. After 15 min incubation, the reaction was terminated by the addition of concentrated H_2_SO_4_. The final chromogen was read at 450 nm on a BioTek plate reader (VT, USA). TPSA was calculated from the calibration curve.

### Parathyroid hormone (PTH) and vitamin D

PTH and vitamin D levels were estimated by ELISA techniques using Sunlong Biotech Co. Ltd. (Hangzhou, China). In brief, microtiter plates coated with PTH and vitamin D antibodies were used. PTH and vitamin D from the blood samples reacted with the antibodies immobilized on the plate and the immunogen was further developed using a second antibody-horseradish peroxidase conjugate. The final chromogen was read at 450 nm.

### Calcium/Magnesium/Phosphorous determination

Serum concentration of Ca, Mg, phosphorous (P), urea, creatinine, sodium, potassium and albumin were determined by standard analytic methods on the Mindray BS 300 Chemistry analyzer (New York, USA) using commercial kits from Elitech (France). All assays were performed with standards and controls.

### Trace element analysis of phytotherapeutic drug

It has been suggested that Mg and other trace elements (TE) supplementation may play a role in shrinking an enlarged prostate. To this end, TE analysis was performed to ascertain whether the phytotherapeutic drug could possible play the role of a TE supplement. In brief, TE were determined by neutron activation analysis (NAA) at the Ghana Atomic Energy Commission. Samples of de-capsulated extract (*Croton membranaceus*) alongside international standards and controls were irradiated in the Ghana Research Reactor – 1 (GHARR-1) operating at 15 KW and a thermal flux of 5 × 1011 n.cm -2. S-1. Samples were irradiated for 5 min followed by 10 min of counting. The radioactivity measurement of induced radionuclide was performed by a PC interfaced with the γ-ray spectrometric equipment. The following trace elements were analyzed: As, Cl, Co, Cr, Cu, Cd, Hg, Pb, Ni, Fe, Mn, I, V, Al, Mg, Se, K, Na, Ca, and Zn

### Statistical analysis

IBM SPSS statistics for Windows, Version 21.0 (Armonk, NY: IBM Corporation) and Microsoft Excel 2013 were used for the statistical analysis. Data was presented as mean ± standard deviation. Statistical analysis between groups was determined by student’s t-test. Categorical variables were expressed as proportion. Correlation analysis was performed with Pearson correlation test. The level of significance was 0.050.

## Results

Mean age for the control group was 56.14 ± 6.77 years and that of the BPH group was 66.13 ± 11.04 years. Mean PSA levels of controls, before treatment (BT) and after treatment cases (AT) were 1.0 ± 0.64, 27.9 ± 19.0 and 16.2 ± 11.8 ng/mL, respectively. There was a significant difference before and after treatment (*P* = 0.002) (Table 1).

**Table 1:** Table showing PSA differences as well as Ca, Mg and PO4 levels of the control and BPH group

	Mean ± SD Control group	Mean ± SD Treatment group (Before –BT)	Mean ± SD Treatment group (After – AT)	*p*-value
PSA ng/mL	1.0 ± 0.64	27.9 ± 19.0 ^b^	16.2 ± 11.8 ^b^	0.002
Mg mmol/L	0.8 ± 0.1 ^a^	0.64 ± 0.18 ^a,b^	0.77 ± 0.16 ^b^	0.0001^a^/0.0031 ^b^
Ca mmol/L	2.30 ± 0.24	2.44 ± 0.85	2.29 ± 0.44	NS
PO_4_ mmol/L	1.4 ± 0.3	1.53 ± 0.35	1.63 ± 0.32	NS
Ca/Mg	2.87 ± 0.48^a^	3.81 ± 1.12 ^a,b^	2.96 ± 0.68 ^b^	0.0001^a^/0.0001 ^b^

^a^ Indicates comparison between control and test groups; whist ^b^ indicates comparison between Before Treatment levels and After Treatment. *NS* not significant (*n* = 30)

Serum Mg concentration was lower for BPH cases (BT = 0.64 ± 0.18, AT = 0.77 ± 0.16 mmol/L), but higher for the control group (0.8 ± 0.1 mmol/L). Control group and BT group Mg differences were significant (*p* = 0.0001). Similarly, Mg BT and AT were different (*p* = 0.0031). Mg concentration differences were no more significantly different after treatment compared to the control or BT (Table 1).

Serum Ca levels did not demonstrate significant differences between the three groups. However, slight increases were observed in the test group before treatment. Although the levels were marginal, levels dropped to that of the control after treatment (C = 2.3 ± 0.24, AT = 2.29 ± 0.44 mmol/L) (Table 1). Similarly, changes in P showed a slight increasing trend from C, BT to AT. However, increases were not significant (Table 1).

The Ca/Mg ratios were as follows: C = 2.87 ± 0.48; BT = 3.81 ± 1.12; AT = 2.96 ± 0.68. Significant differences were observed between the C and BT (*p* = 0.0001) and BT and AT (0.0001). However, the ratio after treatment and that of the control group no longer showed significant differences (Table 1). Parathyroid hormone and vitamin D as well as the renal function test did not show any statistical difference (Tables 2 and 3, respectively).

**Table 2:** Parathyroid hormone (PTH) and Vitamin D levels of the control and BPH group

Parameter	Mean ± SD Control group	Mean ± SD Treatment group (Before - BT)	Mean ± SD Treatment group (After - AT)	*P*-value
PTH (pg/mL)	45.26 ± 19.72	50.68 ± 18.87	49.36 ± 17.61	NS
Vitamin D (ng/mL)	30.60 ± 18.98	28.55 ± 8.70	32.06 ± 12.76	NS

*n* = 30; *NS* not significant

**Table 3:** Renal function tests of the control and BPH group

Parameter	Mean ± SD Control group	Mean ± SD Treatment group (Before- BT)	Mean ± SD Treatment group (After - AT)	*P*-value
Sodium (mmol/l)	140.6 ± 3.5	142.8 ± 6.6	142.2 ± 6.6	NS
Potassium (mmol/l)	4.2 ± 0.5	4.3 ± 0.6	4.3 ± 0.6	NS
Urea (mmol/l)	4.0 ± 2.3	4.3 ± 1.4	4.4 ± 1.3	NS
Creatinine (μmol/l)	89.3 ± 23.4	105.9 ± 26.5	106.0 ± 26.2	NS

*N* = 30; *NS* not significant

There was a positive correlation between Ca and Mg before treatment (*r* = 0.391; *p* = .033). However, this correlation did not exist after treatment with levels similar to the control group. Similarly, there was a positive correlation between Mg and P before treatment (*r* = 0.398; *p* = .029). Correlation no longer existed after treatment with levels similar to the control group (Table 4).

**Table 4:** Pearson’s Correlation analysis

Correlation Parameters		Control Group		BPH Group Before Treatment		BPH Group After Treatment	
Parameter A	Parameter B						
		r	*p*-value	r	*p*-value	r	*p*-value
Ca	Mg	-		.391 ^a^	0.033	-	
Mg	P	-		.398 ^a^	0.029		
Ca	P	-		.529 ^b^	0.003	.523 ^b^	0.003
P	Ca/P	-.884 ^b^	< .001	-.559 ^b^	0.001	-.560 ^b^	0.001
Ca	Ca/Mg	.700 ^b^	< .001	.482 ^b^	0.007	.501 ^b^	0.005
Mg	Ca/Mg	-.755 ^b^	< .001	-.493 ^b^	0.001	-.473 ^b^	0.008
TPSA	Ca/Mg	-		-		-	

^a^ Correlation is significant at the 0.05 level (2-tailed)

^b^ Correlation is significant at the 0.01 level (2-tailed)

Between Ca and P a positive correlation existed in the disease state (Table 4). A phosphate Ca/Mg correlation was highly significant and inversely correlated in the control group (*r* = -0.884). This correlation existed moderately in the test groups (Table 4). Calcium Ca/Mg ratio showed a strong positive correlation in the control group (*r* = 0.700; *p* < .001). This correlation also existed in the disease groups to a moderate extent (Table 4). The magnesium Ca/Mg ratio showed a strong inverse correlation in the control group (*r* = -0.755; *p* < .001). However, the correlation was moderate in the disease group (Table 4). No correlation was observed between TPSA and Ca/Mg ratio.

The prevalence of hypomagnesemia in BPH patients was 86.7% in tandem with a hypercalcemia of 36.7%. Both levels were approximately 5 and 4 times, respectively, that of the control group. After treatment Mg-Ca levels were almost that of the control group (Table 5). The prevalence of the Ca/Mg imbalance using the upper limit of the control group [2.87 (2.39–3.35)] was 80% before treatment (BT). This was reduced to 13.3% after treatment (AT). The control group prevalence was 3.3% (Table 5).

**Table 5:** Prevalence of low Mg and high Ca levels as well as the Ca/Mg ratio imbalance

Prevalence levels of Mg and Ca	Control group	Treatment group (Before – BT)	Treatment group (After – AT)
Prevalence of low Mg (%)	16.7	86.7	13.3
Prevalence of high Ca (%)	10.0	36.7	13.0
Prevalence of Ca/Mg ratio imbalance (%)	3.33	80.0	13.3

*n* = 30

Out of the 30 cases only 18 had their prostate volumes on record. There was no correlation between the Ca/Mg ratio and the prostate volume (Table 6).

**Table 6:** Calcium Magnesium levels and the corresponding prostate volumes of individuals whose data could be retrieved

Patient ID	1	2	3	4	5	6	7	8	9	10	11	12	13	14	15	16	17	18
Ca/Mg ratio	3.11	4.02	4.09	3.82	3.41	3.69	3.41	4.09	3.48	3.15	4.04	3.81	3.60	5.17	3.08	3.46	4.33	5.25
Prostate vol (cm-^3^)	104	15.2	44.4	115	45.8	13.4	131	79.8	137	47.7	94.5	30.1	61	149	49.9	198	150	105

To ascertain whether the Ca/Mg ratio that was apparently corrected after treatment could be related to TE in the phytotherapeutic drug, TE analysis was conducted. From Table 7, Ca was of the highest concentration (58446 mg/kg dry wt.); this was followed by K (7452 mg/Kg dry wt.). The third highest was Mg (3239 mg/kg dry wt.). Traditional antioxidants that have been suggested to aid prostate treatment were very low (Zn = 2.992 and Se = 0.16 mg/Kg dry wt.). Other toxic ultra-trace elements such as Hg, As and Pb were of negligible quantities (Table 7).

**Table 7:** Trace elements in *Croton membranaceus*

Element	Concentration in mg/kg dry wt.
As	0.06
Cl	1608
Co	3.45
Cr	4.13
Cu	<0.003
Cd	<0.002
Hg	<0.001
Pb	<0.001
Ni	<0.001
Fe	19.041
Mn	0.847
I	<0.001
V	3.81
Al	1201
Mg	3239
Se	0.16
K	7452
Na	1128
Ca	58446
Zn	2.992

## Discussion

Minerals are naturally occurring elements found in the earth with a characteristic crystalline structure and chemical composition. Dietary minerals are inorganic nutrients and are usually required in small amounts of 1-2500 mg per day [17]. The inorganic nature of these micro-nutrients differentiates them from the other micro-molecules that are organic such as vitamins [18]. Traditionally, minerals are grouped into two categories; macro (major) - and micro – (trace) elements. A third category is the ultra-trace element [19]. Whereas the macro - and micro - elements are required in amounts > 100 mg/day and < 20 mg/day, respectively, ultra-trace elements are required in the region of 1 mg/day. Arsenic, bromine, boron, chromium, cadmium, fluorine, lead, lithium, molybdenum, nickel, selenium, silicon, tin and vanadium fit into this category [19].

The importance of trace elements to health cannot be under-estimated even though they are required in minute quantities. At least 17 known essential elements and many others are needed for good health and inadequate amounts of trace elements such as sodium, potassium, magnesium and calcium have a link to the development of diseases [20]. Trace metal concentrations including Cd, Ni, Zn, Cu, Fe, Mg, and Ca in both malignant and benign prostate samples have been determined [2].

Imbalance in trace elements such as P, Ca, Mg that are essential to normal human homeostasis, results in a number of clinical complications including prostate cancer. Suggested processes implicated in the progression of prostate disorders are from oxidative stress, to cellular senescence [21, 22]. Mineral metabolism which includes Ca, Mg and P occurs through three main target organs: the bone, intestine and kidney. The homeostatic control of these minerals is primarily facilitated by the parathyroid hormone and vitamin D.. However, there is still a gap in our understanding regarding the relationship between trace element functions and initiation as well as progression and inhibition of the carcinogenic process in the prostatic gland [23, 24].

In this study Mg levels were found to be significantly lower in patients with BPH compared to the control group. Mg has an effect on a variety of cell membranes through a process involving Ca channels and ion transport mechanisms. Mg is therefore responsible for the maintenance of the trans-membrane gradients of sodium and potassium [25].

On the other hand Ca was significantly higher in patients with BPH compared to the controls. Serum Ca was shown not to be associated with the risk of prostate cancer [26]. Contrary to this, a weak negative association has been shown between serum Ca and the incidence of fatal prostate cancer in Swedish men [27]. Furthermore, the association between serum Ca levels and aggressive lesions or fatal prostate cancer has been observed in some studies [28]. It has been previously hypothesized that high serum Ca or PTH increase, is a risk factor for fatal prostate cancer [29]. However, Ca levels in BPH patients have not been previously demonstrated to be associated with the development of this condition.

This study demonstrated a moderate positive association between Ca and Mg as well as Mg and P in the disease state. The association disappeared after treatment and was similar to the status of the controls. Indeed Ca and Mg are both reabsorbed at the loop of Henle. The correlation was perhaps an attempt to create the necessary balance by increasing Mg reabsorption. Although Ca may be reabsorbed alongside Mg, both may not be to the same extent and perhaps Ca reabsorption is truncated when Mg is adequately reabsorbed. It has been shown that Ca reabsorption is not altered in Mg deficiency; however, elevations of extracellular Mg results in a specific inhibition of Ca reabsorption within the loop of Henle [30]. This positive correlation disappeared after treatment when the Ca/Mg ratio had normalized.

The P and Mg positive correlation may be due to P co-absorption with Mg as a compensatory mechanism. The P reabsorption may not be PTH controlled but a reduction in the amount of titrable P at the distal convoluted tubules. It is postulated that an abnormal homeostasis occurs. It has been demonstrated that in parathyroidectomized rats, Mg depletion was equally associated with P depletion. However, one study has suggested that the overall control of renal Mg reabsorption occurs within the loop of Henle and that the proximal tubule reabsorbs a constant fraction of the filtered load despite variations in body Mg status [30]. In that study, proximal Mg reabsorption remained unchanged at 15% of the filtered load and was unaffected by Mg deficiency or acute Mg repletion. However, distal tubular Mg reabsorption was limited during depletion and increased to a similar extent in control and deficient rats with enhanced Mg delivery [30]. The mechanism of P reabsorption under such circumstances of Mg reabsorption is not clearly understood.

Hypomagesemia has been reported in a number of disease conditions diarrhea, vomiting and gastrointestinal fistulas. A prevalence of 11% was reported when 621 hospital patients (in the USA) were randomly selected compared to 2.5% in the control group made up of 341 hospital staff [31]. Other disease conditions such as malabsorption, diarrhea, hypertension, cognitive heart failure, coronary artery disease among others have all been reported to be associated with hypomagnesaemia [32, 33]. Hypomagnesaemia was present in 86.7% of the patients diagnosed with BPH. The control group had a hypomegnesemia prevalence of 16.7% which is comparable to a prevalence of 14.5% among 16,000 apparently normal Germans studied [34]. The prevalence for 11,000 white urban Americans (45 to 64 years) was between 2.5 and 5% depending on the lower limit used. Twice this prevalence was found in African Americans [35]. The 80% prevalence of a high Ca/Mg ratio was reduced to 13.3% after treatment, although this was still higher than the 3.3% prevalence found in the control group.

The phytotherapeutic drug that was administered (made of *Croton membranaceus*) was analyzed for its trace element content. It was noted that the extract was high in Ca, followed by potassium and Mg. Indeed the Ca level was 18 times that of Mg; nevertheless Ca absorption is always tightly regulated. Although the serum Mg level was significantly low while the Ca/Mg ratio was significant high before treatment, the Mg level significantly increased to that of the controls and the Ca/Mg ratio significantly reduced to the level of the controls after treatment.

In several studies investigating the relationship between Ca intake and the risk of developing aggressive or clinically relevant prostate cancers as well as the relationship between prostate disorders, PTH and vitamin D have both generated null [26, 36] and positive results [37]. Blood Ca levels are tightly regulated and only moderately affected by dietary intake of Ca and absorption rates [38]. Thus, one possible explanation for the inconsistencies across study populations is that dietary intake measures of Ca may not accurately reflect the blood Ca concentrations to which prostate tissues are exposed [6].

Whether the phytotherapeutic drug’s trace element levels contributed to the correction of the Ca/Mg imbalance in the BPH group is a mechanism to consider or whether the corrected imbalance is a result of some other mechanism(s), is debatable. The former is very unlikely considering the RDA for Ca and Mg and the overall 60 mg intake of the plant extract drug which is far below the RDA. Nevertheless, the imbalance and its association with the Ca channels remain plausible.

Ca channels and the Ca/Mg ratio is a developing area of focus. The Ca/Mg ratio in this study was significantly raised in the BPH group compared to the control group. Studies have shown that inadequate Mg levels, relative to Ca levels (i.e. high Ca/Mg ratio) are associated with greater risk of prostate disorders. Among high-grade prostate cancer cases compared to controls, serum Mg levels were significantly lower, while the Ca/Mg ratio was significantly higher. An elevated Ca/Mg ratio was therefore associated with an increased risk of high-grade prostate cancer and elevated Mg was significantly associated with a lower risk [6]. However, only one or two studies seem to have examined this phenomenon in BPH patients.

It has been recently demonstrated that a high Ca/Mg ratio is pro-inflammatory and Mg increase reverses this imbalance through gate-keeping activities of TRPM6/7 gene in an apoptotic drive. The apoptotic mechanism of this phytotherapeutic drug made of *Croton membraneus* on BPH-1 cells have recently been demonstrated [39]. Therefore, the present Ca/Mg ratio correction is still in keeping with the apoptotic mechanism established previously by [39].

The PSA levels were also reduced significantly after treatment and symptoms improved significantly by the International Prostate Symptoms Score (IPSS) assessment as published elsewhere [16]. The correlation between Ca/Mg and PSA levels were not established, just as the correlation between prostate volume and the Ca/Mg ratio. Whether this ratio pertains to the initiation or progression of the disease remains unknown and requires further studies.

## Conclusion

Ca/Mg ratio imbalance is associated with BPH similar to observations in PCa. This has previously not been demonstrated. The imbalance was corrected after treatment. Although apoptosis is suspected to be one of the mechanisms for the action of this pytotherapeutic drug, the actual mechanism is yet to be elucidated.

## Abbreviations

AT: After treatment
BPH: Benign prostatic hyperplasia
BT: Before treatment
Ca: Calcium
Mg: Magnesium
NAA: Neutron activation analysis
Pca: Prostate cancer
PSA: Prostate specific antigen
PTH: Parathyroid hormone
RFT: Renal function tests
TE: Trace element
TPSA: Total prostate specific antigen
TRPM: Melastatin-like transient receptor potential

## References

[1] Yang CY, Chiu HF, Tsai SS, Cheng MF, Lin MC, Sung FC (2000). Calcium and magnesium in drinking water and risk of death from prostate cancer. J Toxicol Environ Health A.

[2] Yaman M, Atici D, Bakirdere S, Akdeniz I (2005). Comparison of trace metal concentrations in malign and benign human prostate. J Med Chem.

[3] Tvedt KE, Halgunset J, Kopstad G, Haugen OA (1989). Intracellular distribution of calcium and zinc in normal, hyperplastic, and neoplastic human prostate: X-ray microanalysis of freeze-dried cryosections. Prostate.

[4] Sun Y, Selvaraj S, Varma A, Derry A, Sahmoun AE, Singh BB (2013). Increase in Serum Ca2/Mg2 Ratio Promotes Proliferation of Prostate Cancer Cells by Activating TRPM7 Channels. J Biol Chem.

[5] Lin KI, Chattopadhyay N, Bai M, Alvarez R, Dang CV, Baraban JM, Brown EM, Ratan RR (1998). Elevated extracellular calcium can prevent apoptosis via the calcium-sensing receptor. Biochem Biophys Res Commun.

[6] Dai Q, Motley SS, Smith JA, Concepcion R, Barocas D, Byerly S, Fowke JH (2011). Blood magnesium, and the interaction with calcium, on the risk of high- grade prostate cancer. PLoS One.

[7] Whitfield JF, Boynton AL, MacManus JP, Sikorska M, Tsang BK (1979). The regulation of cell proliferation by calcium and cyclic AMP. Mol Cell Biochem.

[8] Berridge MJ, Brown KD, Irvine RF, Heslop JP. Phosphoinositides and cell proliferation. J. Cell Sci. 1985;(Suppl.3):187–98. 10.1242/jcs.1985.supplement_3.18 3011822

[9] Roderick HL, Cook SJ (2008). Ca2 signaling checkpoints in cancer: remodeling Ca2 for cancer cell proliferation and survival. Nat Rev Cancer.

[10] Sahmoun AE, Singh BB (2010). Does a higher ratio of serum calcium to magnesium increase the risk for postmenopausal breast cancer?. Med. Hypotheses.

[11] Wolf FI, Fasanella S, Tedesco B, Torsello A, Sgambato A, Faraglia B, Palozza P, Boninsegna A, Cittadini A (2004). Regulation of magnesium content during proliferation of mammary epithelial cells (HC-11). Front Biosci.

[12] Rubin H (2005). Central roles of Mg2 and Mg ATP in the regulation of protein synthesis and cell proliferation: significance for neoplastic transformation. Adv Cancer Res.

[13] Hajno’czky G, Davies E, Madesh M (2003). Calcium signaling and apoptosis. Biochem Biophys Res Commun.

[14] Rizzuto R, Pinton P, Ferrari D, Chami M, Szabadkai G, Magalha˜es PJ, DiVirgilio F, Pozzan T (2003). Calcium and apoptosis: facts and hypotheses. Oncogene.

[15] Chubanov V, Waldegger S, Mederos Y, Schnitzler M, Vitzthum H, Sassen MC, Seyberth HW, Konrad M, Gudermann T (2004). Disruption of TRPM6/TRPM7 complex formation by a mutation in the TRPM6 gene causes hypomagnesemia with secondary hypocalcemia. Proc Natl Acad Sci U S A.

[16] Asare GA, Afriyie D, Ngala RA, Annan Y, Appiah AA, Musah I, Adjei S, Bamfo KB, Sule SD, Gyan BA, Ahin P, Edoh DA. Shrinkage of prostate and improved quality of life: management of BPH patients with croton membranaceus ethanolic root extract. Evid Based Complement Alternat Med. 2015:10. Article ID 365205. 10.1155/2015/365205 PMC4461762 26106434

[17] Soetan KO, Olaiya CO, Oyewole OE (2010). The importance of mineral elements for humans, domestic animals and plants: a review. Afr J Food Sci.

[18] Blake JS, Munoz KD, Volpe S (2013). Nutrition: from science to you.

[19] Nielsen FH (1993). Ultratrace elements of possible importance for human health: an update. Prog Clin Biol Res.

[20] Sánchez-Benito JL, Sánchez-Soriano E, Ginart SJ (2007). Unbalanced intake of fats and minerals associated with risk hypertension by young cyclists. Nutr Hosp.

[21] Begley L, Monteleon C, Shah RB, Macdonald JW, Macoska JA (2005). CXCL12 overexpression and secretion by aging fibroblasts enhance human prostate epithelial proliferation in vitro. Aging Cell.

[22] Bethel CR, Chaudhary J, Anway MD, Brown TR (2009). Gene expression changes are age-dependent and lobe-specific in the brown Norway rat model of prostatic hyperplasia. Prostate.

[23] Guntupalli JNR, Padala S, Gummuluri AVRM, Muktineni RK, Byreddy SR, Sreerama L, Kedarisetti PC, Angalakuduru DP, Satti BR, Venkatathri V, Pullela VBRL, Gavarasana S (2007). Trace elemental analysis of normal, benign hypertrophic and cancerous tissues of the prostate gland using the particle- induced X-ray emission technique. Eur J Cancer Prev.

[24] Banas A, Kwiatek WM, Banas K, Gajda M, Pawlick B, Cichocki T (2010). Correlation of concentrations of selected trace elements with Gleason grade of prostate tissues. J Biol Inorg Chem.

[25] Bara M, Guiet-Bara A, Durlach J (1993). Regulation of sodium and potassium pathways by magnesium in cell membranes. Magnes Res.

[26] Halthur C, Johansson AL, Almquist M, Malm J, Grönberg H, Manjer J, Dickman PW (2009). Serum calcium and the risk of prostate cancer. Cancer Causes Control.

[27] Van Hemelrijck M, Wigertz A, Sandin F, Garmo H, Hellström K, Fransson P, Widmark A, Lambe M, Adolfsson J, Varenhorst E, Johansson J-E, Stattin P (2013). Cohort profile: the national prostate cancer register of Sweden and prostate cancer data base Sweden 2.0. Int J Epidemiol.

[28] Skinner HG, Schwartz GG (2008). Serum calcium and incident and fatal prostate cancer in the National Health and Nutrition Examination Survey. Cancer Epidemiol Biomarkers Prev.

[29] Skinner HG, Schwartz GG (2009). The relation of serum parathyroid hormone and serum calcium to serum levels of prostate-specific antigen: a population-based study. Cancer Epidemiol Biomarkers Prev.

[30] Carney SL, Wong NL, Quamme GA, Dirks JH (1980). Effect of magnesium deficiency on renal magnesium and calcium transport in the rat. J Clin Invest.

[31] Wong ET, Rude RK, Singer FR, Shaw ST (1983). A high prevalence of hypomagnesemia and hypermagnesemia in hospitalized patients. Am J Clin Pathol.

[32] Topf JM, Murray PT (2003). Hypomagnesemia and hypermagnesemia. Rev Endocr Metab Disord.

[33] Dacey MJ (2001). Hypomagnesemic disorders. Crit Care Clin.

[34] Schimatschek HF, Rempis R (2001). Prevalence of hypomagnesemia in an unselected German population of 16,000 individuals. Magnes Res.

[35] Ma J, Folsom AR, Melnick SL, Eckfeldt JH, Sharrett AR, Nabulsi AA, Hutchinson RG, Metcalf PA (1995). Associations of serum and dietary magnesium with cardiovascular disease, hypertension, diabetes, insulin, and carotid arterial wall thickness: the ARIC study. Atherosclerosis Risk in Communities Study. J Clin Epidemiol.

[36] Chan JM, Stampfer MJ, Ma J, Gann PH, Gaziano JM, Giovannucci EL (2001). Dairy products, calcium, and prostate cancer risk in the Physicians’ Health Study1,2,3. Am J Clin Nutr.

[37] Tseng M, Breslow RA, Graubard BI, Ziegler RG (2005). Dairy, calcium, and vitamin D intakes and prostate cancer risk in the National Health and Nutrition Examination Epidemiologic Follow-up Study cohort. Am J Clin Nutr.

[38] Iseri LT, French JH (1984). Magnesium: nature’s physiologic calcium blocker. Am Heart J.

[39] Afriyie DK, Asare GA, Bugyei K, Lin J, Peng J, Hong Z (2015). Mitochondria- dependent apoptogenic activity of aqueous root extract of *Croton membranaceus* against human BPH-1 cells. Gen Mol Res.

